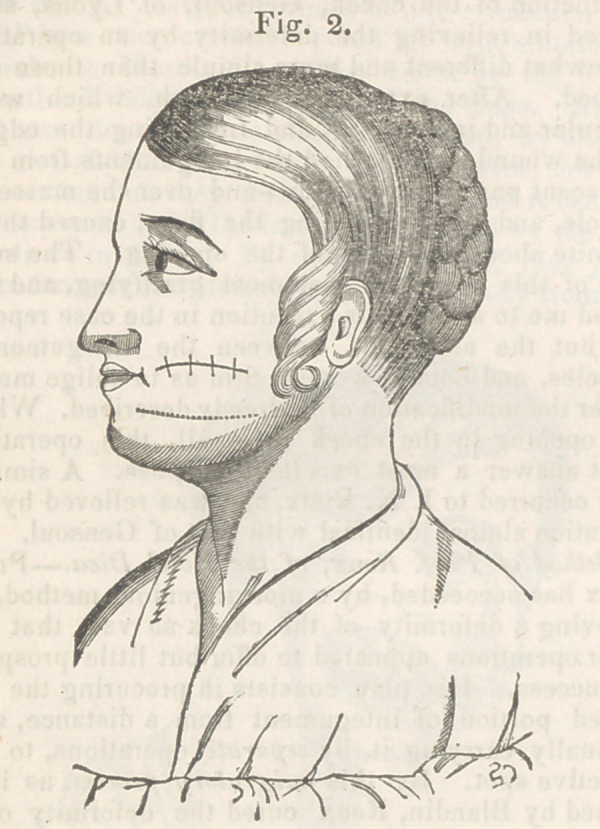# Extensive Meloplastic Operation

**Published:** 1844-05-18

**Authors:** Thomas D. Mütter

**Affiliations:** Professor of Surgery in Jefferson Medical College, &c.


### EXTENSIVE MELOPLASTIC OPERATION.

BY THOMAS D. MÜTTER, M. D.,

Professor of Surgery in Jefferson Medical College, &c.

In the month of March, 1842, A. T———, aged 30, of Clearfield county, Pennsylvania, applied to me for the relief of a distressing deformity, occasioned by the abuse of mercury. About six years before I saw her, she had been most severely salivated for bilious fever; and, in consequence of ulceration attacking the right cheek, nearly the whole of this portion of the face was destroyed. The extensive loss of substance is well represented in fig. 1. To conceal the deformity, she has been in the habit of keeping her face "tied up" in a handkerchief; con-

Fig. 1.

sequently, but little motion being allowed the lower jaw, this partial rest of the organ, persevered in for more than six years, has produced a permanent contraction of the masseter muscles on each side, so that scarcely any motion exists in the temporo-maxillary articulations, and it is impossible to introduce any substance more than the sixth of an inch in thickness between the upper and lower jaw. Her speech is, of course, very much impaired, and all her food is reduced to the smallest possible bulk, or taken in the shape of liquids. Her general health is excellent.

The first indication in such a case was, obviously, to obtain as much motion in the articulations of the lower jaw as possible; and this could only be accomplished by increasing the space between the maxillary bones. To accomplish this, it was deemed best to divide the masseter muscles, (the entire muscle on the left, and what remained of it on the right side,) and then separate the bones by a lever of some kind. Accordingly, on the first Wednesday in March, that being the regular clinical day at the College, she was brought before the class, and the operation performed with a common scalpel, the muscles being divided from *within*, and the edge of the knife carried obliquely downwards and outwards. The wounds were dressed with dry lint, and, on the second day, the lever of Heister was employed to separate the jaws. Each day the screw was turned a thread or two; and, after the lapse of two weeks, the patient was enabled to protrude her tongue without difficulty,—a thing utterly impossible when the treatment was commenced,—and the space between the teeth, when the lower jaw is depressed, is nearly an inch. She has, of course, free motion in the part, and chews her food without much difficulty.

The most difficult part of the treatment still remained to be accomplished; and on Wednesday, the 23d inst., she was again brought before the class, for the purpose of having this put into execution.

After carefully considering the different operations usually performed in such cases, I adopted the following plan:—Having first extracted the useless teeth of the upper jaw, which, from their irregularity, would have materially interfered with the proper adjustment of the flaps, and, besides, by their sharpness, possibly caused ulceration and sloughing of the tissues forced against them, I proceeded to detach the integuments by which the opening in the cheek was surrounded. The edge of the scalpel was directed towards the bone, and the incisions carried sufficiently far to allow the margins of the wound to be approximated to a considerable degree. This callous margin, formed of the "inodular tissue," was then carefully pared off with a bistoury, in order to obtain, if possible, union by the "first intention" between the edges of the flaps. An effort was then made to close the wound, by sliding the detached integuments, from all sides, towards the centre, but they refused to yield, and it became nsecessary to make the incisions indicated by the dotted lines in fig. 2. By these incisions, *four flaps* were formed, and detaching them carefully from the

Fig. 2.

subjacent parts, we found no difficulty in uniting them at a line which indicated the longest diameter of the opening. The twisted suture was employed, and the wound presented, after their introduction, the appearance exhibited in fig. 3. To support the whole, one or two straps were passed over the points

Fig. 3.

upon which their was most strain, and over all a thin pledget of patent lint was laid, and the patient placed in bed. The hemorrhage was comparatively trifling, but few arteries requiring the ligature; and the operation, though painful and tedious, was borne by the patient without a murmur.

24th. Patient passed a good night; has no fever, but slight headache, and warm surface. The wound is cool, and but slightly tumified; bowels not opened. Ordered an enema of salt and water, &c., and no food or drink to be taken. Of course no attempt at speaking has been allowed.

25th. Patient more comfortable; skin moist; no fever; thirst; enema had operated well; allowed to swallow a mouthful or two of water.

26th. Removed the top dressings, and found the flaps cool and united perfectly, with the exception of an opening, about the size of a small shot, in the centre of the cheek. General condition of the patient same as on the 25th. Ordered gruel and cool water every hour or two, and also an enema, as the bowels were not opened the day before.

28th. Removed needles; parts adhered, except just at the centre of the wound.

30th. Touched the edges of the orifice with argent. nit., and applied a cerate cloth.

Simple dressings, with the application of the caustic, were continued for several days, but the little wound refused to contract or granulate; and I therefore freshened the edges with the scalpel, and drew them together with a twisted suture. Union, by this plan, was speedily accomplished, and my patient relieved of a most shocking deformity.

Fig. 4.

REMARKS.

There is probably no defect, for the removal of which "plastic surgery" is required, more difficult to remedy than an extensive opening in the cheek. On this point, Dieffenbach, Blandin, Roux, Liston, Zies, and, indeed, all surgeons who have directed their attention to this department of surgery unite in opinion. To Delpech and Lallemand the credit of being the *first* to make an attempt at relieving the deformity is usually rendered; although Franco, in all probability, is better entitled to it. Several operations have been devised for the defect in question; but it must be obvious that, while certain *general* rules of action may be laid down, no one series of details will answer in every case.

*Lallemand's Method.*—The plan usually resorted to in cases of partial destruction of the cheek, unless the opening is very small, is that proposed by Lallemand. In this operation, after having first freshened the edges of the wound, a flap is taken from the adjacent integument of the neck, *turned upon its entire pedicle*, by which means torsion is obviated, and then attached by the twisted suture to the margins of the wound it is intended to occupy. The accompanying figures, taken from one of my cases, illustrate the steps of this operation better than language can describe them.

Fig. 1.

Fig. 2.

In Lallemand's case, there was much difficulty experienced, from the restive disposition of the child, but the operation eventuated successfully. From the fact that, in this method, the base of the flap is subjected to very slight torsion,—the great obstacle to success in most cases of plastic surgery,—it has found many advocates, and is to be preferred, in my opinion, whenever practicable, to any other.

*Dupuytren's Method.*—Dupuytren, in cases similar to the above, was in the habit of taking his flap from the most convenient parts, but often *twisted it upon its base, as is done* in some forms of the Rhinoplastic operation; and, according to his statement, with the most perfect success. There is more danger of sloughing, of course, when the flap is subjected to torsion, and, although the method has been followed by successful results, yet it should never be employed when the operation of Lallemand can be carried into effect.

*Gensoul's Method.*—In a case of most extensive destruction of the cheek, Gensoul, of Lyons, succeeded in relieving the deformity by an operation somewhat different and more simple than those described. After extracting the teeth, which were irregular and in the way, and freshening the edges of the wound, he detached the integuments from the subjacent parts above, below, and over the masseter muscle, and then, by sliding the flaps, caused them to unite about the centre of the opening. The success of this operation was most gratifying, and induced me to attempt its execution in the case reported, but the adhesions between the integuments, muscles, and bones, were so firm as to oblige me to prefer the modification of it already described. When the opening in the cheek is small, this operation must answer a most excellent purpose. A similar case occurred to I. N. Roux, and was relieved by an operation almost identical with that of Gensoul.

*Method of Prof. Roux, of the Hôtel Dieu.*—Prof. Roux has succeeded, by a most ingenious method, in relieving a deformity of the cheek so vast that all other operations appeared to offer but little prospect of success. His plan consists in procuring the required portion of integument from a distance, and gradually carrying it, by *separate* operations, to the defective spot. By this *migratory process*, as it is termed by Blandin, Roux cured the deformity of a girl who had lost a portion of the left side of the upper lip, the corresponding ala of the nose, and part of the cheek. The flap was taken from the lower lip, and first attached to the upper, and then subsequently transferred to the cheek. The patient was under treatment a year, and submitted to several *severe operations*.

*Method of Dieffenbach.*—In those cases where the flaps are made to approach each other without difficulty, Dieffenbach, to relieve them from the strain, and thus obviate the danger of separation of the wound after the sutures are withdrawn, has been in the habit of making an incision across the base of the flap, as first advised for *other operations*, in which the parts are too tense, by Thévenin.

In the case from which the following drawings

Fig. 1.

were taken, I adopted the plan of Dieffenbach in part, and with the most decided benefit. After freshening the edges of the wound, I drew them together, and made the incision indicated by the dotted line in fig. 2. All strain was thus taken off the flap; and, inasmuch as this was attached by its extremities,

Fig. 2.

and could thus be well supplied with blood, I made the cut as soon as the wound in the cheek was closed.

My operation in the first case, differs, in many respects, from those just described, although it resembles, somewhat, that of Gensoul; but future repetition must prove whether or not it is to be preferred.

## Figures and Tables

**Fig. 1. f1:**
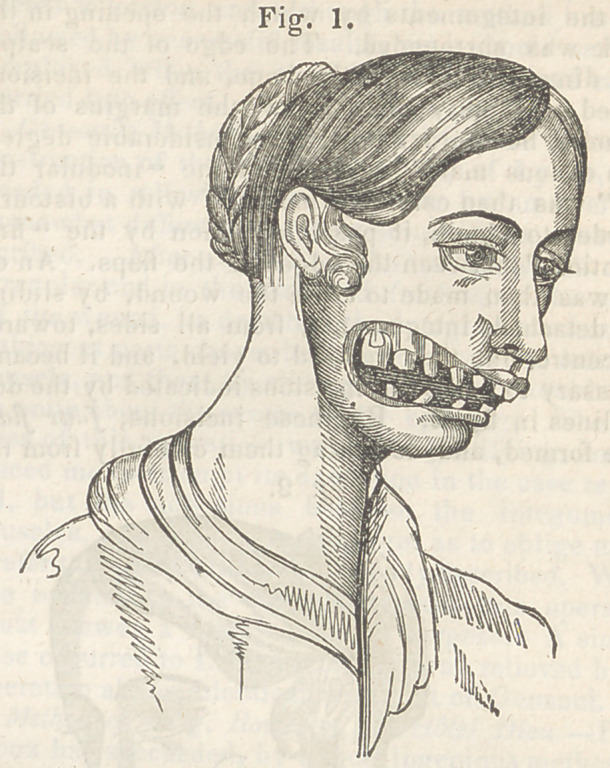


**Fig. 2. f2:**
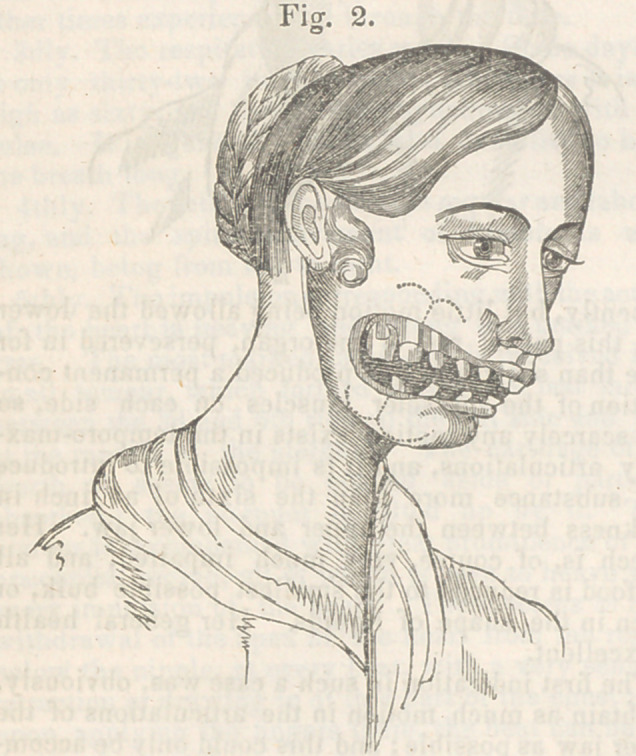


**Fig. 3. f3:**
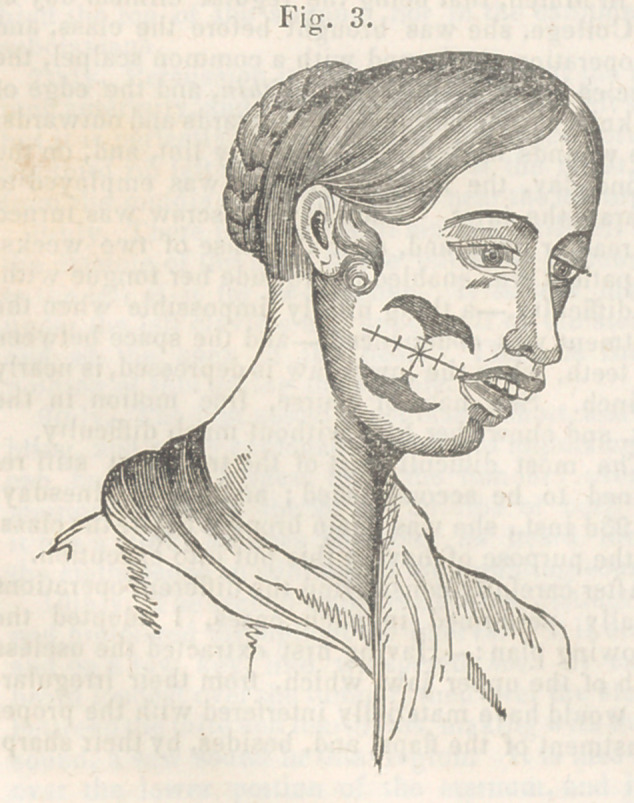


**Fig. 4. f4:**
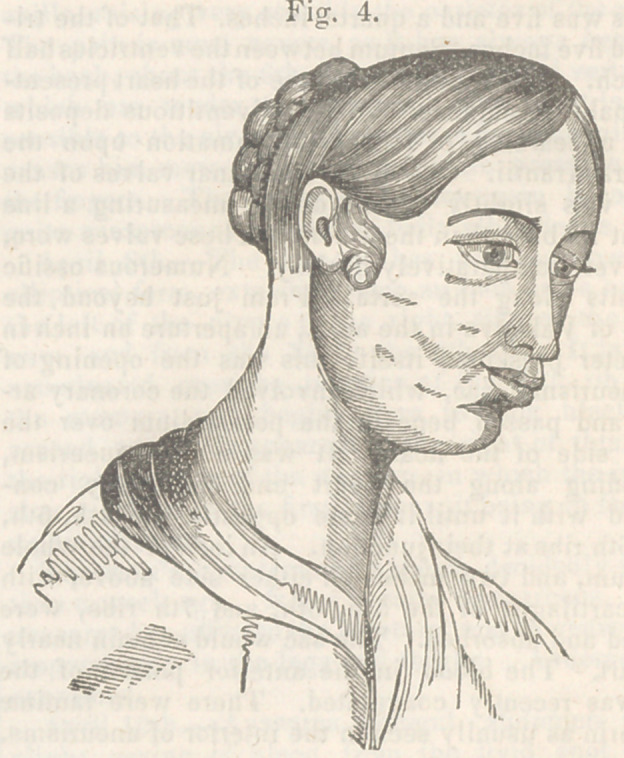


**Fig. 1. f5:**
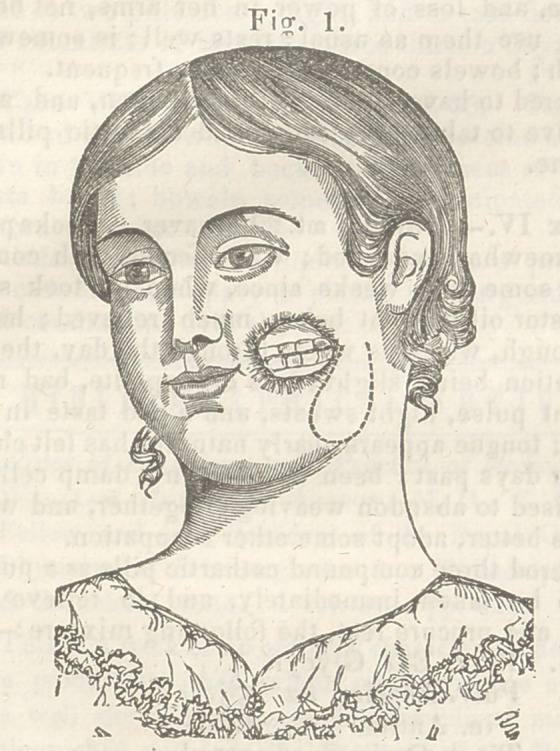


**Fig. 2. f6:**
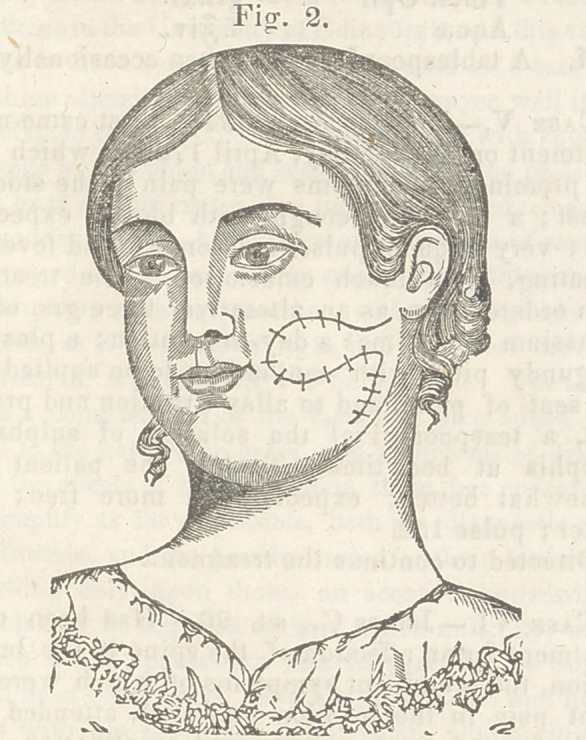


**Fig. 1. f7:**
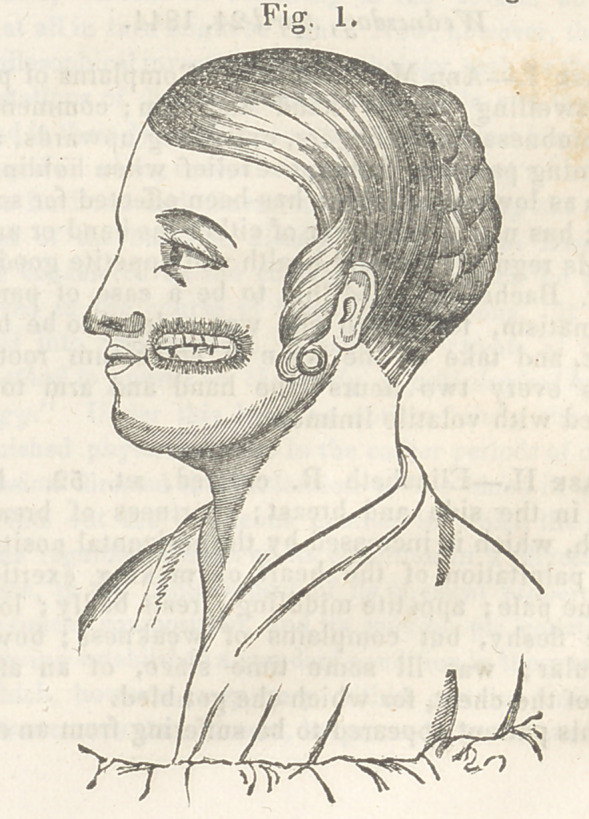


**Fig. 2. f8:**